# Evaluation of factors associated with medication adherence in patients with bipolar disorder using a medication event monitoring system: a 6‐month follow‐up prospective study

**DOI:** 10.1186/s12991-022-00411-4

**Published:** 2022-08-23

**Authors:** HyunChul Youn, Moon-Soo Lee, Hyun-Ghang Jeong, Seung‑Hyun Kim

**Affiliations:** 1 Department of Psychiatry, Soonchunhyang University Bucheon Hospital, Bucheon, Republic of Korea; 2 Department of Child and Adolescent Psychiatry, Korea University Guro Hospital, Seoul, Republic of Korea; 3 Korea University Research Institute of Mental Health, Seoul, Republic of Korea; 4 Department of Psychiatry, Korea University Guro Hospital, Korea University College of Medicine, 148, Gurodong-ro, Guro-gu, Seoul, 08308 Republic of Korea

**Keywords:** Bipolar disorder, Adherence, Compliance, Mania, Anticonvulsants, Weight gain

## Abstract

**Background:**

Non-adherence in patients with bipolar disorder (BD) results in symptoms, such as aggravation, BD recurrence, emergency room visits, re-hospitalization, and poor psychosocial outcomes. Though non-adherence rates have been reported to range between 30–50% in patients with BD, the problem of adherence is often either overlooked by the physician or denied by the patient. An essential first step to enhancing medication adherence is to objectively estimate adherence. The Medication Event Monitoring System (MEMS), which is a pill bottle cap with a microprocessor, is an accurate device for assessing medication adherence. Using the MEMS, we aimed to measure medication adherence in patients with BD and evaluate the factors associated with and 6-month changes in medication adherence.

**Methods:**

Participants with BD were recruited from the psychiatric outpatient clinic of the Korea University Guro Hospital. The medication adherence of each participant was assessed using the MEMS, a self-report, pill count, and clinician rating. MEMS-measured adherence was reassessed after 6 months. Patient demographics were recorded and clinical assessments were conducted. Data were analyzed using Kappa statistics and Pearson’s correlation analysis.

**Results:**

Of the 59 participants, 50 records were included in the analysis. Patient adherence and adherence rate assessed by the MEMS were lower than those assessed by the other measures. MEMS-measured adherence was correlated more closely with pill counts than with self-reports or clinician ratings. MEMS-measured adherence was negatively associated with prescription duration and the Brief Psychiatric Rating Scale—Affect Subscale Score. Six-month changes in MEMS-measured adherence were positively associated with attitude toward drugs and negatively associated with weight gain assessed by the Udvalg for Kliniske Undersøgelser Side Effect Rating Scale.

**Conclusions:**

Clinicians may have to consider the limited accuracy of self-reporting and clinician rating methods and exercise caution when assessing the medication adherence of patients with BD using these methods. Our findings may assist clinicians in the assessment and improvement of medication adherence in patients with BD and, consequently, may be useful for the treatment and prevention of BD recurrence.

## Background

Bipolar disorder (BD) refers to a recurrent mood disorder characterized by episodes of hypomania or mania and depression interspersed with periods of euthymia [1]. BD has been shown to affect 1–2% of the general population; however, some researchers have reported a prevalence of 4–5% [2, 3]. BD can impair quality of life and social functioning, and its substantial socioeconomic burden impacts patients, their families, and society [2]. It is also associated with high rates of mortality, suicide, and medical comorbidities [4]. The World Health Organization reported that, because of its early onset and chronicity across the lifespan, BD contributes to the loss of disability-adjusted life-years more than Alzheimer’s disease, cancer, or epilepsy [5]. A study by Forte et al. indicated that the proportion of time spent ill because of BD constituted more than 40% of the lifespan [6].

Treatment with medications, such as lithium and anticonvulsants, is the primary treatment option for patients with BD [7, 8], and the issue of medication adherence has received much clinical attention. Several studies have reported the problem of low adherence in patients with BD [7, 9–11]. Although non-adherence rates tend to vary across study settings, they have been reported to range between 30–50% [12–14]. Non-adherence in patients with BD results in symptoms, such as aggravation, BD recurrence, emergency room visits, re-hospitalization, and poor psychosocial outcomes [15, 16]. An essential first step to enhancing medication adherence is to objectively estimate adherence.

Patient adherence can be characterized as “the extent to which a patient’s behavior coincides with the medical advice the person has received [17].” Various methods have been implemented to measure medication adherence. The self-report method is one of the most common methods for measuring adherence; however, it can be biased by memory deficits, the level of disease severity, denial, or mimicking good adherence [18]. The clinician’s report has also often been used to measure medication adherence. This method may also be biased because it may be based on the self-report method [18]. Pill counts and plasma level measurements are relatively objective methods; however, these methods have certain limitations. Pill counts may be an ambiguous way to measure medication adherence because they cannot discriminate between good adherence and alternating over- and under-adherence, missing pills, or discarding of pills. Moreover, plasma levels have often been used to assess adherence in patients with BD who take lithium or anticonvulsants; however, these assessments may be influenced by inter- and intra-patient variability and may not accurately represent medication adherence [18]. To more accurately and objectively measure medication adherence, some studies have adopted the medication event monitoring system (MEMS; Aprex Corporation, Fremont, CA, USA) to assess patients with medical or psychiatric disorders [18–23]. The MEMS is a pill bottle cap that contains a microprocessor, which tracks a patient’s usage of the medication bottle [23]. The MEMS has been recognized as a relatively accurate method for assessing medication adherence [24].

Sajatovic et al*.* compared the MEMS with the self-report method in patients with BD and found that the MEMS identified 20% more non-adherence than the self-report method [21]. However, few studies have used the MEMS to assess medication adherence in patients with BD, and compared medication adherence measured by the MEMS with that measured by other methods [21]. Thus, our study sought to measure MEMS adherence in patients with BD and compare it with patient adherence assessed by the self-report, pill count, and clinician rating methods. We hypothesized that the medication adherence recorded by the MEMS may be lower than that assessed by other measures, as postulated by Sajatovic et al*.* [21]. Several factors affecting medication adherence in patients with psychiatric disorders include attitude toward medication, insight, therapeutic alliance, illness duration, social support, and life circumstances [25–30]. Previous BD studies have reported that attitude toward illness, health beliefs, personality, alcohol or other substance abuse, and medication side effects were associated with medication non-adherence [12, 31–34]. Therefore, this study also evaluated factors associated with MEMS-measured adherence in patients with BD, which may help clinicians enhance medication adherence in patients with BD. For this analysis, we included BD history, sociodemographic, and clinical variables. Furthermore, by re-evaluating MEMS-measured adherence after 6 months, we prospectively assessed changes in long-term medication adherence and maintenance-related factors, which are particularly important in patients with BD.

## Methods

### Participants

We recruited participants with BD from the psychiatric outpatient clinic of the Korea University Guro Hospital, Seoul, Republic of Korea. Our patient inclusion criteria were as follows: (1) aged 18–65 years; (2) met the diagnostic criteria for BD specified in the *Diagnostic and Statistical Manual for Mental Disorders, 5*^*th*^* edition* [35]; (3) took medications for BD, including lithium or anticonvulsants; and (4) had unchanged BD medication dosages for at least 2 weeks. We applied the following patient exclusion criteria: (1) had any disease resulting in cognitive dysfunction (e.g., intellectual disabilities); (2) had alcohol or other substance use disorders, or (3) were acutely suicidal. The recruitment process, including the diagnosis of patients, was conducted by board-certified psychiatrists.

### Procedure

This study measured MEMS adherence two times at 6-month intervals. For the 6-month follow-up study, we recruited volunteers from the initial participants because some patients did not want to participate in the long-term follow-up study and were excluded. At enrollment, all participants were provided with either lithium or anticonvulsants in a bottle with the MEMS. If a participant was prescribed more than one tablet of lithium or anticonvulsants per day, then only one tablet per day was added to the MEMS bottle. The MEMS follow-up duration was either each participant’s routine visit interval or 1 month. Patient demographics were recorded and clinical scale measurements were conducted at enrollment. At the second visit, the medication adherence of each participant was assessed using the MEMS and other adherence measures. Then, we estimated the MEMS adherence of each participant after 6 months. The study protocol was approved by the Institutional Review Board of the Korea University Guro Hospital (2011GR0021). All participants provided written informed consent to participate in the study.

### Measurement

Patient demographics, including age, sex, education level, and marital, housing, and occupational status were obtained from an interview, a questionnaire, and chart record review. Each participant’s BD information and history were also investigated. Moreover, we measured the height, weight, and body mass index of each patient at the first and second visits.

#### Clinical scales

The Clinical Global Impressions-Severity (CGI-S) scale was used to assess current psychopathological severity [36]. The CGI-S scale is a clinical rating scale that is scored from 1 (*not ill*) to 7 (*severely ill*). The Brief Psychiatric Rating Scale (BPRS) is a clinical rating scale based on semi-structured interviews assessing various psychiatric symptoms [37]. The BPRS contains 18 items on a scale of 0 (*not present*) to 6 (*extremely severe*). Shafer indicated five BPRS domains, including affect, positive symptoms, negative symptoms, resistance, and activation [38]. The present study used these subscales. The Young Mania Rating Scale (YMRS) and Hamilton Rating Scale for Depression (HAM-D) are clinical rating scales composed of 11 and 17 items, respectively [39, 40]. We used the YMRS to estimate manic symptoms and the HAM-D to measure depressive symptoms. The Multidimensional Scale of Perceived Social Support (MSPSS) is a self-rating scale [41], and we used it to evaluate the perceived social support of family, friends, and significant others. For the MSPSS, each domain contains four items that are rated on a seven-point scale ranging from “very strongly disagree” (1) to “very strongly agree” (7). The Drug Attitude Inventory (DAI) is a self-rating scale that was used to assess attitudes toward psychotropic medications [42]. The DAI contains ten dichotomous items that can be classified into positive and negative subjective feelings. The Mood Disorder Insight Scale (MDIS) is an eight-item questionnaire comprising three subscales, including awareness of illness, attribution, and need for treatment [43]. We adopted this self-rating scale to measure the degree of patient insight. Additionally, this study included the Udvalg for Kliniske Undersøgelser Side Effect Rating Scale (UKU-SERS) to evaluate the side effects of psychopharmacological medications [44]. The reliability and validity of all of these scales have been confirmed by previous studies [36, 37, 39–44].

#### Adherence measures

The primary outcome measure of this study was MEMS-measured adherence. With the MEMS, adherence is recorded as long as the participant opens the bottle within a 3-h target time frame. From the data retrieved from the MEMS cap, we obtained the percentage of doses taken on schedule (i.e., [number of doses taken correctly according to the prescription/number of prescribed doses] × 100). We subtracted the MEMS-measured adherence at the second visit from that at 6 months and defined this value as the “6-month change in MEMS-measured adherence”. We also checked the self-report adherence by asking the participants to estimate their adherence to the prescribed BD medications on a scale of 0–100%. In addition, pill count adherence (i.e., percentage of the actual pill count/prescribed pill count) was calculated. To assess pill count adherence, a clinician counted all of the remaining prescribed BD medication pills that the participants had at the second visit. The clinician rating scale of adherence is a 1–7 scale, with higher scores indicating better adherence [19]. Clinician rating assessment was conducted by a clinician who was not aware of the MEMS cap data. The results from the MEMS, self-report, and pill count methods were categorized into adherence and non-adherence based on an 80% threshold [18, 23], whereas a score of ≥ 5 on the clinician rating scale indicated acceptable adherence [19].

### Statistical analysis

Descriptive statistics were calculated for all variables, with percentages calculated for categorical variables and means and standard deviations calculated for continuous variables. We calculated Kappa statistics to assess the degree of agreement between the dichotomized MEMS-measured adherence and adherence assessed by other measures [45]. Associations between MEMS-measured adherence or 6-month changes in MEMS-measured adherence and other variables were also evaluated using Pearson’s correlation analysis. A *p*-value of < 0.05 was considered statistically significant. All statistical analyses were performed using PASW Statistics 18.0 for Windows (SPSS Inc., Chicago, Illinois).

## Results

**Fig. 1:**
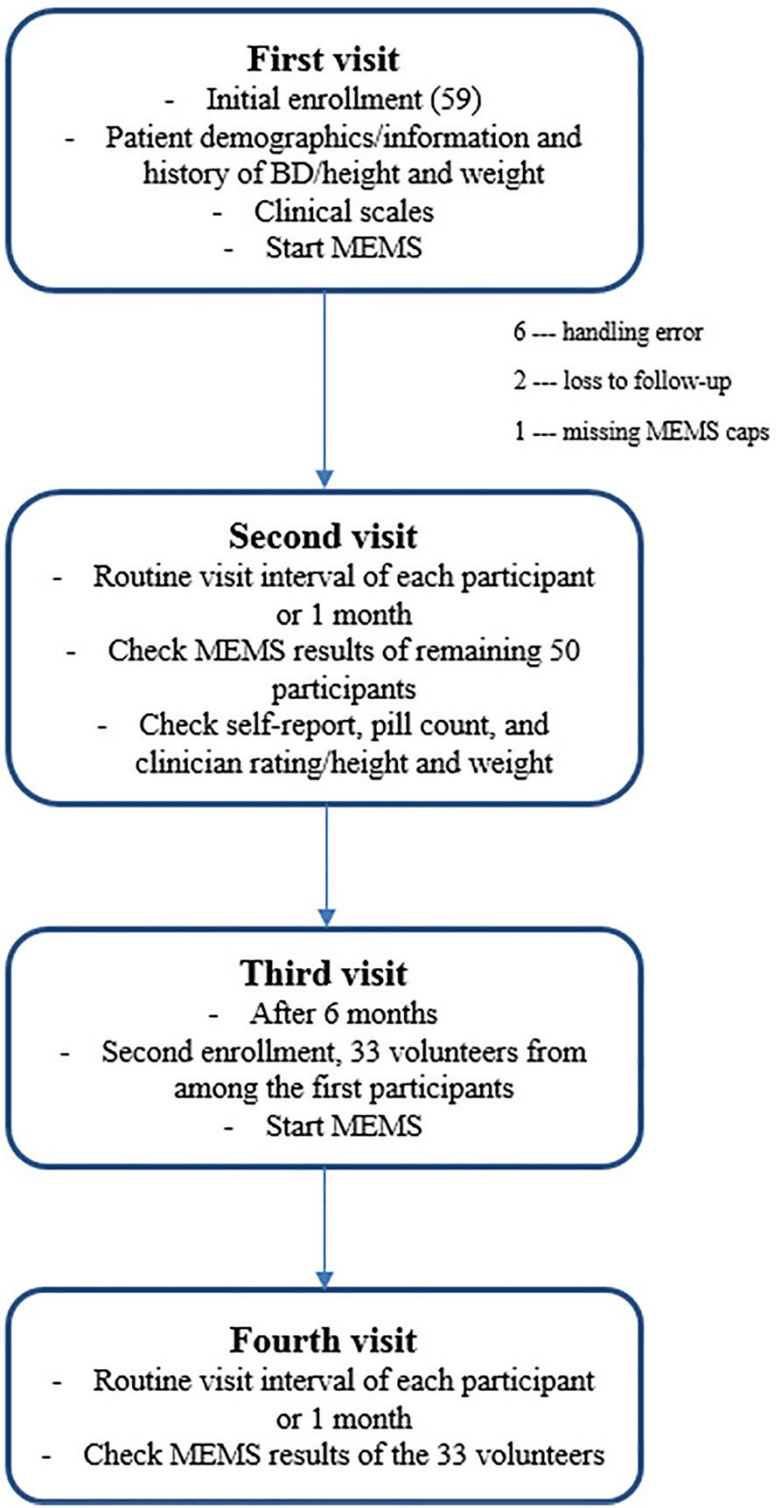
Study process. *BD* bipolar disorder, *MEMS* medication event monitoring system

The study process is shown in Fig. 1. Initially, we recruited 59 participants. Of these patients, 13 were provided with lithium, 43 with valproic acid, and three with other anticonvulsants. At the second visit, nine participants were excluded due to handling errors—keeping the cap open for a long time/taking out a large amount of medication tablets at once and taking it over several days—a missing MEMS cap, or loss to follow-up. Therefore, this study included the MEMS records of the remaining 50 participants with BD. The mean age of the participants was 36.50 (*SD* = 13.82) years. Table 1 provides the participant demographics.

**Table 1:** Participant demographics (*n* = 50)

Characteristics	Categories	*n* (%) or mean ± SD
Age (years)		36.50 ± 13.82
Sex	Male	24 (48)
	Female	26 (52)
Education (years)		13.54 ± 2.72
Marital status	Single	29 (58)
	Married/living together	16 (32)
	Separated/divorced	5 (10)
Housing status	Live alone	4 (8)
	Live with family	41 (82)
	Other	5 (10)
Occupation	Unemployed/stay-at-home spouse	26 (52)
	Student	11 (22)
	Employed	13 (26)
Baseline weight (kg)		65.87 ± 13.00
Weight change (kg)		2.97 ± 2.61
Baseline BMI		23.65 ± 3.83
BMI change		1.07 ± 0.96

*SD* standard deviation, *BMI* body mass index

The mean adherence values for each adherence measurement method are as follows: MEMS, 85.69%; self-report, 91.76%; and pill count, 92.16%. For the clinician rating scale of adherence, the mean score was 5.10 (*SD* = 0.58). The mean 6-month change in MEMS-measured adherence was −4.78 (*SD* = 31.69). Six-month changes in MEMS-measured adherence were calculated using MEMS data from 33 patient volunteers. In our study, 44 (88%) participants had experienced a recent manic episode, and the mean YMRS and HAM-D scores were 6.60 (*SD* = 7.48) and 8.68 (*SD* = 7.6), respectively. Table 2 provides descriptive statistics for the adherence and clinical variables.

**Table 2:** Adherence and clinical variables of participants (*n* = 50)

Characteristics	Categories	*n* (%) or mean ± SD
Adherence measures	MEMS	85.69 ± 18.52
	6-month changes in MEMS-measured adherence	−4.78 ± 31.69
	Self-report	91.76 ± 12.15
	Pill count	92.16 ± 13.04
	Clinician rating	5.10 ± 0.58
Duration of illness (months)		67.50 ± 80.20
Number of recurrent episodes		2.34 ± 2.20
Number of hospital admissions		2.24 ± 1.83
Recent episode	Manic episode	44 (88)
	Depressive episode	6 (12)
Number of total medication tablets per day		7.99 ± 4.59
Prescription duration (days)		38.88 ± 36.52
CGI-S score		2.84 ± 1.06
BPRS scores	Affect	5.04 ± 4.17
	Positive symptoms	3.30 ± 4.64
	Negative symptoms	2.36 ± 2.53
	Resistance	2.26 ± 3.50
	Activation	2.34 ± 2.40
	Total score	15.30 ± 12.82
YMRS score		6.60 ± 7.48
HAM-D score		8.68 ± 7.65
MSPSS score		60.92 ± 14.07
DAI score		3.40 ± 4.24
MDIS score		8.94 ± 2.32
UKU-SERS scores	Sleepiness, sedation	0.59 ± 0.79
	Weight gain	0.42 ± 0.76

*SD* standard deviation, *MEMS* medication event monitoring system, *CGI-S* Clinical Global Impressions-Severity, *BPRS* Brief Psychiatric Rating Scale, *YMRS* Young Mania Rating Scale, *HAM-D* Hamilton Depression Rating Scale, *MSPSS* Multidimensional Scale of Perceived Social Support, *DAI* Drug Attitude Inventory, *MDIS* Mood Disorders Insight Scale, *UKU-SERS* Udvalg for Kliniske Undersøgelser Side Effect Rating Scale

In addition, continuous adherence data were dichotomously coded (i.e., adherent vs. non-adherent). From this data, the calculated adherence rates for the MEMS, self-report, pill count, and clinician rating methods were 78.0, 90.0, 86.0, and 94.0%, respectively. We also assessed the degree to which the MEMS-measured adherence agreed with the adherence measured by the other measures following dichotomization. The Kappa coefficient between the MEMS and pill count methods exhibited the highest level of agreement (*κ* = 0.598, *p* < 0.001; Table 3) according to the guidelines proposed by Landis and Koch in which coefficients ranging from 0.61–0.80 are substantial and those ranging from 0.41–0.60 are moderate [45].

**Table 3:** Kappa coefficient between the MEMS and other adherence measures

	Kappa coefficient	*p*-value
MEMS/self-report	0.275	0.031^*^
MEMS/pill count	0.598	< 0.001^**^
MEMS/clinician rating	0.054	0.625

*MEMS* medication event monitoring system

^*^p < 0.05; ^**^p < 0.01

From Pearson’s correlation analysis, MEMS-measured adherence was found to be negatively associated with prescription duration (*r* = *−*0.321, *p* = 0.023) and the BPRS—Affect Subscale Score (*r* = *−*0.349, *p* = 0.013), whereas the 6-month changes in MEMS-measured adherence had significant associations with the weight gain item of the UKU-SERS (r = −0.537, *p* = 0.005) and total DAI scores (r = 0.392, *p* = 0.024; Tables 4, 5).

**Table 4:** Pearson correlation coefficients for MEMS-measured adherence and other variables

	MEMS-measured adherence (first measurement)	*p*-value
Age (years)	0.257	NS
Education (years)	−0.042	NS
Baseline weight (kg)	−0.108	NS
Weight change (kg)	0.176	NS
Baseline BMI	−0.059	NS
BMI change	0.170	NS
Duration of illness (months)	0.109	NS
Number of recurrent episodes	0.177	NS
Number of hospital admissions	0.041	NS
Number of medication tablets per day	0.024	NS
Prescription duration (days)	−0.321	0.023^*^
CGI-S score	−0.127	NS
BPRS scores		
Affect	−0.349	0.013^*^
Positive symptoms	−0.068	NS
Negative symptoms	0.045	NS
Resistance	−0.256	NS
Activation	−0.240	NS
Total score	−0.244	NS
YMRS score	−0.070	NS
HAM-D score	−0.119	NS
MSPSS score	−0.029	NS
DAI score	−0.179	NS
MDIS score	−0.079	NS
UKU-SERS scores		
Sleepiness, sedation	−0.089	NS
Weight gain	−0.098	NS

*MEMS* medication event monitoring system, *NS* not significant, *BMI* body mass index, *CGI-S* Clinical Global Impressions-Severity, *BPRS* Brief Psychiatric Rating Scale, *YMRS* Young Mania Rating Scale, *HAM-D* Hamilton Depression Rating Scale, *MSPSS* Multidimensional Scale of Perceived Social Support, *DAI* Drug Attitude Inventory, *MDIS* Mood Disorders Insight Scale, *UKU-SERS* Udvalg for Kliniske Undersøgelser Side Effect Rating Scale

^*^p < 0.05; ^**^p < 0.01

**Table 5:** Pearson correlation coefficients for 6-month changes in MEMS-measured adherence and other variables

	6-month changes in MEMS-measured adherence	*p*-value
Age (years)	−0.078	NS
Education (years)	−0.009	NS
Baseline weight (kg)	0.094	NS
Weight change (kg)	0.008	NS
Baseline BMI	0.005	NS
BMI change	−0.014	NS
Duration of illness (months)	−0.144	NS
Number of recurrent episodes	−0.261	NS
Number of hospital admissions	0.081	NS
Number of medication tablets per day	−0.082	NS
Prescription duration (days)	0.236	NS
CGI-S score	0.333	NS
BPRS scores		
Affect	0.316	NS
Positive symptoms	−0.033	NS
Negative symptoms	−0.025	NS
Resistance	0.122	NS
Activation	0.161	NS
Total score	0.144	NS
YMRS score	0.154	NS
HAM-D score	0.283	NS
MSPSS score	−0.134	NS
DAI score	0.392	0.024^*^
MDIS score	0.124	NS
UKU-SERS scores		
Sleepiness, sedation	−0.112	NS
Weight gain	−0.537	0.005^**^

*MEMS* medication event monitoring system, *NS* not significant, *BMI* body mass index, *CGI-S* Clinical Global Impressions-Severity, *BPRS* Brief Psychiatric Rating Scale, *YMRS* Young Mania Rating Scale, *HAM-D* Hamilton Depression Rating Scale, *MSPSS* Multidimensional Scale of Perceived Social Support, *DAI* Drug Attitude Inventory, *MDIS* Mood Disorders Insight Scale, *UKU-SERS* Udvalg for Kliniske Undersøgelser Side Effect Rating Scale

^*^p < 0.05; ^**^p < 0.01

## Discussion

In this study, adherence assessed by the MEMS was lower than adherence measured by self-reporting and pill counts. Similarly, when the adherence data were dichotomized, the adherence rate measured by the MEMS was lower than those assessed by the other adherence measures. The pill count method exhibited a higher degree of agreement with the MEMS method compared with the other adherence measures. MEMS-measured adherence was negatively associated with prescription duration and the BPRS—Affect Subscale Score. Six-month changes in MEMS-measured adherence were positively associated with attitude toward drugs, whereas a negative association was found between 6-month changes in MEMS-measured adherence and weight gain assessed by the UKU-SERS.

In this study, the observed MEMS-measured adherence was higher than that reported by other MEMS studies on depression and schizophrenia, which used similar methods [18, 23]. Our MEMS-measured adherence result was also higher than that reported by Sajatovic et al*.*, who studied patients with BD on a broad episode spectrum [21]. The majority of our participants had experienced a recent manic episode (88%). Manic symptoms may be severe and dramatic for patients and their caregivers. We speculate that this may be related to the relatively high degree of adherence observed in this study. Although few studies have reported an association between the occurrence of recent manic episodes and medication adherence, Gonzalez-Pinto et al*.* investigated the adherence of 1831 patients who had experienced recent manic or mixed episodes and reported a similar adherence level to that of our study [46].

Consistent with our hypothesis, MEMS monitoring reported a greater number of non-adherent participants than the other adherence measures. In addition, the self-report and clinician rating methods detected non-adherence with limited accuracy, as demonstrated by their Kappa coefficients, whereas the pill count method demonstrated a relatively high level of agreement with the MEMS. These results suggest that clinicians may have to consider the possibility of overestimation when they use the self-report, pill count, or clinician rating methods to assess medication adherence in patients with BD. Of these non-MEMS methods, the pill count method may more accurately reflect medication adherence than the self-report and clinician rating methods.

This study also evaluated the factors associated with MEMS-measured adherence in patients with BD. Increased prescription duration was associated with decreased adherence. The prescription duration reflects this study’s MEMS follow-up duration, which was defined as each participant’s routine visit interval or 1 month. Therefore, this suggests that increased periods between hospital visits were related to decreased adherence. We speculate that the associated issues of forgetfulness and carelessness over time may have influenced our results. Therefore, when medication non-adherence is suspected, clinicians may have to increase the frequency of follow-up visits for patients with BD.

The BPRS—Affect Subscale Score also exhibited a negative correlation with medication adherence. The BPRS—Affect Subscale Score includes items on anxiety, guilt, depression, and somatic symptoms [38]. Our result was consistent with those of previous studies. Belzeaux et al*.* investigated the self-reported adherence of 382 patients with BD and reported that residual depressive, but not manic symptoms, were the main factors associated with adherence behavior [47]. Montes et al*.* demonstrated that recent depressive polarity was associated with low medication adherence [48]. Gutiérrez-Rojas et al*.* also reported that a high frequency of depressive episodes was related to poor medication adherence [49]. Although few studies have investigated the cause of this association, cognitive symptoms, such as attention or memory disturbances, and lack of motivation that can occur during the depressive state may affect medication adherence [47]. Our study results suggest that the presence of residual depressive symptoms must be taken into consideration to improve medication adherence in patients with BD.

Furthermore, we assessed 6-month changes in MEMS-measured adherence and analyzed the factors associated with the observed changes. In this study, the 6-month changes in MEMS-measured adherence showed a negative value (−4.78), suggesting that the medication adherence of patients with BD may tend to decrease over time. Extra care should be taken to assure long-term adherence. A decrease in adherence at 6 months was associated with a negative attitude toward drugs and weight gain assessed by the UKU-SERS. Previous studies have reported positive associations between attitude toward drugs and adherence [50–52]. Our analysis confirmed that this association might apply to 6-month changes in adherence. Weight gain in patients with BD is 20–35% more prevalent than that in the general population [53–55]. Pharmacotherapy is the major cause of the high prevalence of weight gain in patients with BD [56]. The negative correlation between weight gain and adherence is generally well known and should be considered by clinicians [12, 31, 34, 57]. The UKU-SERS is a clinician-rated scale based on a semi-structured interview [44]. Specifically, the weight gain item of the UKU-SERS assesses the subjective thoughts of participants about their weight gain. Therefore, perceived weight gain rather than real weight gain was associated with the 6-month MEMS-measured adherence changes observed in our study. This suggests that clinicians may have to consider the issue of medication adherence in patients who complain of subjective weight gain, regardless of objective weight gain.

The DAI reflects subjective thoughts about medication, such as the necessity of drugs and relaxed, tired, and “doped up” feelings [42]. Weight gain assessed by the UKU-SERS reflects the perceived side effects of psychopharmacological medication [44]. Therefore, the attitude toward drugs and perceived weight gain associated with 6-month MEMS-measured adherence changes may be factors that relate to the medication itself. Specifically, the results of this study suggest the importance of medication-specific approaches for maintaining long-term medication adherence. Therefore, it may be necessary to thoroughly discuss patients’ thoughts and feelings about medications and their side effects when assessing patients with BD. Furthermore, clinicians may have to promote medication understanding and solve medication-associated problems. Previous studies have reported the effectiveness of psychoeducation programs, including the explanation of psychopharmacotherapy, in enhancing medication adherence [33, 58, 59]. Such programs might also help maintain long-term medication adherence in patients with BD.

There are some limitations to this study. First, this study had a relatively small number of participants. In addition, the participants were only enrolled from a university hospital. Thus, it is necessary to be careful when considering the generalizability of our results. Furthermore, due to the nature of this study that required operating the device, patients with cognitive impairment were included in the exclusion criteria, and participants with handling errors were excluded from the analysis. Considering that many patients with BD suffer from cognitive impairment, this may also be a limitation in generalizing this study [60]. Second, the participants recognized that this study sought to estimate adherence. This might have encouraged greater adherence during the study period. Third, the study participants may have been subject to selection bias, with non-adherent individuals refusing to participate or being lost to follow-up. Additionally, although the recruitment process was conducted by board-certified psychiatrists, it is possible that subjective views may have influenced the application of inclusion and exclusion criteria. Fourth, although patients with cognitive impairment were included in the exclusion criteria, this study did not conduct a thorough evaluation of patients' ability to express consent or to conduct the study. Considering that the populations of bipolar patients, even in the euthymic phase, have varying degrees of ability to express consent, it may be necessary to carry out a preliminary assessment of the ability to express consent to the study [61]. Likewise, if the patient's ability to conduct the study had been checked more closely, the number of dropouts due to handling error could have been reduced. Fifth, we did not specifically classify other medications such as antipsychotics taken in conjunction with lithium or anticonvulsants, though this study adopted number of total medication tablets per day as a variable. We believe that applying a more specific medication classification would be helpful in future studies. Sixth, the MEMS recorded the act of opening the bottle as adherence. However, the act of bottle opening does not necessarily indicate that the medication or correct dose was taken. Finally, only one drug type (i.e., lithium or an anticonvulsant) was added to the MEMS bottle because the MEMS cannot automatically differentiate between drug types. Therefore, in cases in which the participants arbitrarily took only some of the prescribed medications on time, MEMS-measured adherence might be inaccurately reflected.

This study investigated the medication adherence of patients with BD using the MEMS and compared the findings with the adherence measured by the self-report, pill count, and clinician rating methods. Our results suggest that the self-report and clinician rating methods may report inaccurate and overestimated levels of adherence. Therefore, clinicians may have to consider the limited accuracy of these methods and exercise caution when assessing the medication adherence of patients with BD using these methods. This may be particularly important when considering treatment options, such as dose modification or switching to or adding another medication. In addition, we analyzed the factors associated with MEMS-measured adherence. Prescription duration and the presence of residual depressive symptoms were notable factors associated with MEMS-measured adherence. These factors can be readily evaluated in clinical practice through a chart review or brief questionnaire. Therefore, these findings may help clinicians identify non-adherent patients with BD who require additional attention. Furthermore, the patient’s attitude toward drugs and perceived weight gain were associated with long-term (i.e., 6-month) changes in adherence. These factors were different from those associated with initial MEMS-measured adherence. This difference suggests that specific approaches may have to be implemented to maintain long-term medication adherence in patients with BD. Many previous studies have reported the importance of maintaining pharmacotherapy in patients with BD. Thus, many clinicians have focused on the long-term medication adherence of patients with BD [8, 62]. Our findings may be a useful reference for clinicians aiming to improve long-term medication adherence in patients with BD.

## Conclusions

In conclusion, the MEMS revealed lower adherence and adherence rate levels than other measures, particularly the self-reporting and clinician rating methods. Prescription duration and the presence of residual depressive symptoms were factors associated MEMS-measured adherence, whereas 6-month changes in MEMS-measured adherence exhibited significant correlations with attitude toward drugs and perceived weight gain.

These findings may assist clinicians in the assessment and enhancement of medication adherence in patients with BD and, consequently, may be useful for the treatment and prevention of BD recurrence.

## Data Availability

The datasets used and/or analyzed during the current study are available from the corresponding author on reasonable request.

## Abbreviations

BD: Bipolar disorder
BPRS: Brief Psychiatric Rating Scale
CGI-S: Clinical Global Impressions-Severity
DAI: Drug Attitude Inventory
HAM-D: Hamilton Rating Scale for Depression
MDIS: Mood Disorder Insight Scale
MEMS: Medication event monitoring system
MSPSS: Multidimensional Scale of Perceived Social Support
UKU-SERS: Udvalg for Kliniske Undersøgelser Side Effect Rating Scale
YMRS: Young Mania Rating Scale
